# A Combined Non-Invasive Approach to the Study of A Mosaic Model: First Laboratory Experimental Results

**DOI:** 10.3390/jimaging5060058

**Published:** 2019-06-10

**Authors:** Antonina Chaban, Vivi Tornari, Rita Deiana, Michalis Andrianakis, David Giovannacci, Vincent Detalle

**Affiliations:** 1 Department of Cultural Heritage, Archaeology and History of Art, Cinema and Music, University of Padova, Piazza Capitaniato 7, 35139 Padova, Italy; 2 Institute of Electronic structure and Laser, Foundation for Research and Technology Hellas, N. Plastira 100, Voutes, 70013 Crete, Greece; 3 Laboratoire de Recherche des Monuments Historiques, Ministère de la Culture et de la Communication, 29 rue de Paris, 77 420 Champ-sur-Marne, France; 4 Sorbonne Universités, Centre de Recherche sur la Conservation (CRC, USR 3224), Muséum national d’Histoire naturelle, Ministère de la Culture et de la Communication, CNRS, CP21, 36 rue Geoffroy-Saint-Hilaire, 75005 Paris, France; 5 C2RMF, Centre de Recherche et de Restauration des Musées de France, Palais su Louvre, Porte des Lions, 14 quai François Mitterrand, 750001 Paris, France

**Keywords:** mosaic, interferometry, holography, thermography

## Abstract

This paper presents first laboratory results of a combined approach carried out by the use of three different portable non-invasive electromagnetic methods: Digital holographic speckle pattern interferometry (DHSPI), stimulated infrared thermography (SIRT) and holographic subsurface radar (HSR), proposed for the analysis of a custom-built wall mosaic model. The model reproduces a series of defects (e.g., cracks, voids, detachments), simulating common deteriorated, restored or reshuffled areas in wall mosaics. DHSPI and SIRT, already well known in the field of non-destructive (NDT) methods, are full-field contactless techniques, providing complementary information on the subsurface hidden discontinuities. The use of DHSPI, based on optical imaging and interferometry, provides remote control and visualization of surface micro-deformation after induced thermal stress, while the use of SIRT allows visualization of thermal energy diffusion in the surface upon the induced thermal stress. DHSPI and SIRT data are complemented by the use of HSR, a contact method that provides localized information about the distribution of contrasts in dielectric permittivity and related possible anomalies. The experimental results, made by the combined use of these methods to the identification of the known anomalies in the mosaic model, are presented and discussed here as a contribution in the development of an efficient non-invasive approach to the in-situ subsurface analysis of ancient wall mosaics.

## 1. Introduction

The use of the mosaics as a type of wall decoration is documented in ancient times, since the Roman period [1,2,3]. Mosaics became the main wall decoration of the churches in the Byzantine art, with a large spread in the early Christian age and during the Medieval period [4,5]. After the decline of Byzantine mosaic art in the 13th century, this kind of wall decoration, along with wall paintings, recovers its importance during the Renaissance period [1,2,3]. Occasional uses of mosaics in walls, vaults, and cupolae are documented up to the 19th century in Europe, as in Russia, while in the Orient the use of the mosaics in walls decoration is widely spread during the millennia in the Islamic art, in particular in the Iranian decoration history [1,2,3,6]. Undoubtedly, considering the large use of the wall mosaics in historical buildings in high and not easily accessible parts (e.g., vaults or cupolae), the preservation of these decorations requires the adoption of the most adequate strategies for their quick monitoring and intervention, if needed. 

Wall mosaics are generally made by assembling small, normally cubic pieces (tesserae) of different materials (e.g., coloured glass, stone, nacre) to compose polychrome pictorial representations or geometrical patterns [7,8]. During history, different execution techniques of the mosaic substrate were used. A number of studies were carried out on historical wall mosaics, related to the early Christian, Byzantine and Medieval periods, which contributed to the knowledge about the hidden layers below the decorated surfaces [9,10,11]. These wall mosaics usually contained three substrate layers, obtained by a mix of lime with marble powder. The application mode of the mortar could be different. In order to secure even better fixing of the layers, the first and the second layer had a rough surface, in the form of deep bumps or ripples made by the tip of the trowel. Some nails or hooks were often placed, in order to furtherly secure the placement of mortar and the connection of the substrate to the bricks of the wall [1,2,3,11,12].

The main alteration and degradation phenomena that affect the mosaics include detachments of tesserae, loss of adhesion between layers, presence of moisture, deterioration, and damages in the subsurface and supporting structure [7,13].

Some recent experimental studies are focused on the development of non-invasive integrated approaches to the in-situ analysis of wall decorations [13,14,15,16,17]. However, in our awareness, the new developments of non-invasive scientific methods are not always involved in real conservation practices. In this sense, the development of an efficient integrated non-invasive approach could be crucial for the study of wall mosaics. 

From the consideration of typical degradation and alteration phenomena encountered in historical wall mosaics, it is clear that main conservation issues (degradation phenomena and possible previous restorations) refer to the surface or to the shallow subsurface below the tesserae. 

The possibility to quickly detect the presence of these anomalies using some portable electromagnetic methods, used in the field of non-invasive analysis, offers undoubtedly a new way for the monitoring of wall mosaics.

Through a series of implemented laboratory tests, a combination of electromagnetic non-invasive methods is evaluated here both in terms of their capability to detect hidden defects (detachments, voids, cracks, etc.) and to respond to most common mosaic conservation problems and in terms of their applicability to a wall mosaic surface, operating depth, reliability of obtained information and their resolution. 

## 2. Materials and Methods 

### 2.1. Sample Description

**Figure 1:**
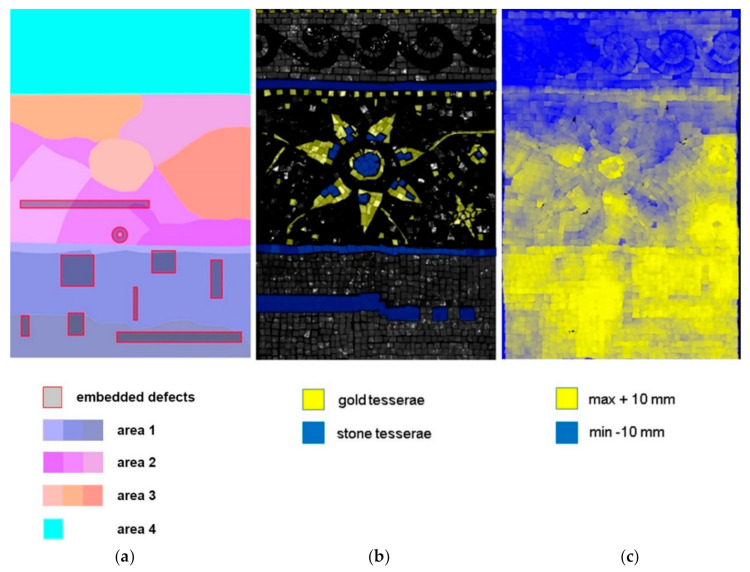
The structure of the mosaic sample: (**a**) Position of the embedded defects (highlighted by the red contour) and structure of the mortar (different colours correspond to different portions of mortar, which vary in proportions of the ingredients and application mode). The separately applied portions are indicated by different colour brightness. The area 1 consists of three to four homogeneous and accurately applied mortar layers (mixture ratio 4:2:1). The area 2 consists of three inhomogeneous layers and the area 3 consists of one to two very inhomogeneous and quickly applied mortar portions (mixtures ratio 4.25:2:1, 4:2.25:1 and 4:2:1.25). The area 4 consists of one thin homogeneous mortar layer (mixture ratio 4:2:1), (**b**) scheme of golden (yellow) and stone (blue) tesserae, (**c**) topography of the surface, obtained by 3D optical scanner survey. The map of colour gradients shows the highest points on the surface in yellow and the lowest points in blue.

**Figure 2:**
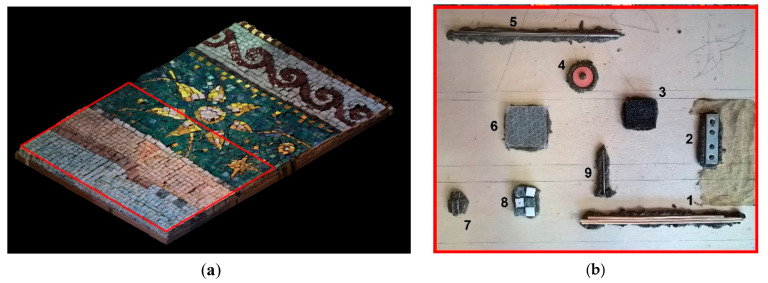
Defects inside the mortar subsurface structure: (**a**) 3D virtual model of the mosaic, the red rectangle indicates the area with the embedded defects, (**b**) photographic documentation of the defects: (1) Three wooden sticks (diameter 3 mm, length 180–200 mm each), (2) one steel inox plate with four holes (15 × 60 × 1 mm), (3) one semi-rigid synthetic sponge (35 × 35 × 5 mm), (4) one rubber ring, with a central hole (diameter 25 mm, thickness 3 mm), (5) one metallic stick (diameter 5 mm, length 200 mm), (6) air bubble wrap (45 mm width × 50 mm length, thickness 1–5 mm), (7) one metallic clip, steel wire (30 × 10 mm × 1 mm), (8) three marble and three glass tiles (around 10 × 10 × 8 mm each), (9) one steel nail (diameter 2 mm, head 5 mm, length 45 mm).

The laboratory experiments were carried out on a custom-built mosaic model. It was realized in collaboration with the mosaic workshop of the Angelo Orsoni Foundry [18] and consists of three main parts: (a) Support, (b) substrate, (c) tesserae. A plywood sheet of the dimensions 400 × 600 × 5 mm was used to provide the structural support for the model transportation. Taking in consideration the variety of discontinuities and defects that can be found in the subsurface layer of a real ancient mosaic decoration, we used not a simplified but rather a complex model (Figure 1), with known defects and discontinuities at the surface and subsurface levels. These include different materials for tesserae, surface irregularity, embedded small elements, simulating defects, and a variable mortar structure (Figure 1 and Figure 2).

In particular, glass paste, golden leaf, and stone tesserae were used for the decoration, in order to test the application of methods to different surface materials, found in real cases [19,20] (Figure 1b). The decoration layer consists of three types of tesserae: (1) Manually cut glass paste tesserae of the Angelo Orsoni Foundry [18,19], (2) manually cut golden leaf tesserae (cartellina) [1,2,3,19] of the Angelo Orsoni Foundry [18,19], (3) manually cut stone tesserae from Italian quarries: white Carrara, red Verona marble and grey basalt.

Cement based mortar inlays of the substrate were prepared separately and applied differently. The proportions of ingredients (cement powder, acrylic glue, and water) and the application mode (covered area, roughness, speed and quantity of substrate layers) were designed in a way to simulate possible inhomogeneity inside the historical mosaics: Eventual consolidation injections, rearrangements and mosaic integrations. The mortar portions were prepared using following proportions of the ingredients: The most used mixture for this mosaic model was 4:2:1 (cement Maurer 86248 Edilbriko, water, acrylic glue ratio), with variations ranging from 4.25:2:1 (maximum ratio for cement), 4:2.25:1 (maximum ratio for water), 4:2:1.25 (maximum ratio for acrylic glue). The application of mortar was erformed in the following ways: (1) Regular mode, consisting of three homogeneous mortar layers, corresponding to the bottom part of the mosaic layout, (2) irregular mode, consisting of several inhomogeneous inlays of mortar portions in the central part of the mosaic layout, (3) regular homogeneous mortar layer in the upper part of the mosaic layout. 

Therefore, the thickness of the mosaic model varies, with the maximum difference in height at the decorated surface of 20 mm. The visual representation of the model surface irregularity using three-dimensional scanning survey and is shown in Figure 1c.

The embedded metallic elements of known thickness, form, and size simulate the presence of fixation elements and joints underneath the decoration. Mosaic tiles represent residues of plastered mosaic tesserae (stone and glass) underneath. Thin wooden sticks are the elements that can be found inside the historical Byzantine mosaic substrate layers [1,2,3,11]. The air bubble wrap represents a loss of adhesion between layers (a shallow detachment), while a semi-rigid synthetic sponge (predominantly air) simulates a bigger detachment. The presence of plastic in the wrap and in the sponge was necessary for the formation of the defect with the necessary thickness and its persistence after the mortar setting down. A shallow rubber ring represents a non-original material with very different dielectric and thermal properties if compared to the surrounding medium, as an indicator of a strong anomaly. The defects were placed upon the first bedding layer, 5–7 mm thick, as shown in Figure 2b. Their location depth is in the range of 15–20 mm under the surface level and is illustrated in Table 1.

### 2.2. Methodology and Experimental Procedure

#### 2.2.1. Holographic Subsurface Radar 

**Figure 3:**
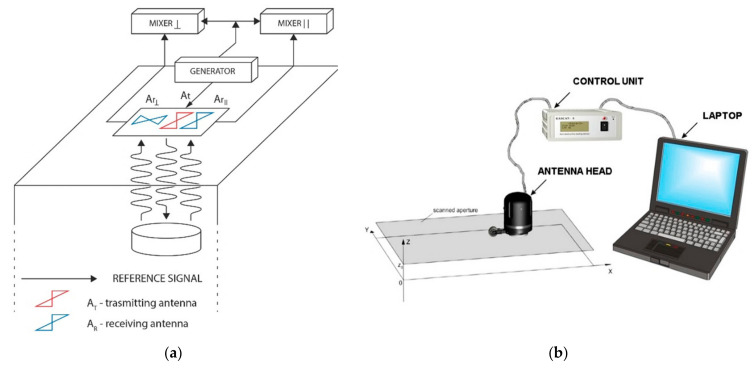
Operating principle (**a**) and operating scheme (**b**) of the holographic subsurface radar system, after Vladimir Razevig [27].

Holographic subsurface radars (HSR), selected for this study, are innovative systems that operate with continuous narrowband, using several frequencies from 2 GHz up to 7 GHz, (depending upon the model [21,22,23,24,25,26,27]), and based on interference techniques for the image formation. Their well-known applications include structural and historical building surveys, non-destructive testing of dielectric materials, security and humanitarian demining [21,22,23]. The detectability of defects in the subsurface by the means of HSR is based on contrasts in dielectric permittivity between the target and the medium. Therefore, the system is effective in the detection of hidden metallic, plastic elements under the planar surfaces, as well as in the detection of moisture [24,25]. In our awareness, this system is still not commonly involved in the field of cultural heritage diagnostics. The fundamental difference of the holographic subsurface radar from the other surface penetrating radar systems [28] is that it provides not a profile but plan-view subsurface images. HSR uses signal processing methods analogous to optical hologram technology, which was first proposed and accomplished by Gabor in 1948 [29]. The relative dimensions of the recording systems constitute the main difference between optical holography and subsurface radar holography. For optical holography, a size-to-wavelength ratio of *d*/*λ* ≅ 10^6^ applies (where *λ* is the wavelength, *d* is a representative dimension of the system), while the same parameter for HSR is equal to only a few units [27]. The laws of geometrical optics cannot be simply applied for HSR implementation. Nevertheless, the analogy is critical for the understanding of the physical processes of HSR and for interpretation of HSR holograms. This technique can also be referred to as microwave holography [30]. Reflected signals are mixed with an internally generated reference signal that has not been transmitted to the medium (Figure 3a). Both the reflected signal and reference signal have the same frequency, but the reflected signal has a phase shift depending on the distance (depth) to the reflector. Thus, the reflected signal and reference signal interfere constructively or destructively depending on the target or the depth of its position. The phase shift of the reflected signal depends on the distance to the reflector, which allows the estimation of the defect depth. More details on the hologram formation can found in the relative publications [21,22,23,24,25,26,27]. 

The holographic subsurface radar, used in this study, consists of a transmitting antenna and two receiving antennas for parallel and cross polarizations, as shown in Figure 3a. It emits unmodulated, continuous wave signals with frequency of 6.4–6.8 GHz [26]. The system operates at five simultaneous frequencies within the microwave range in order to ensure that a target (contrast in dielectric properties) will be visible at least at one of the frequencies, regardless of the depth of its location. In the case of only one frequency used, the so-called “blind depths” may appear for a particular target depth due to sinusoidal variation with depth in the phase difference between the reference and target beams [27]. These five frequencies are automatically switched during scanning and their values are very close (within 10%) to the nominal frequency. After each acquisition, 10 images are obtained (five frequencies, two antenna polarizations) [26,27].

The HSR results are visualized as “in-plane” horizontal maps of the intensity of the waves [21,22,23,24,25,26,27], which contain a sum of reflections from different depth levels. Switching the five discrete frequencies, when the dielectric permittivity of the constituent material is known, it is possible to focus the image at the specific depth by the means of the dedicated software. When focusing at a specific depth, the strongest response is expected to derive from the selected level. The image, however, still contains the sum of reflections that come from different depths. For more detailed information about the HSR system, the reader is asked to consult the relative literature references [21,22,23,24,25,26,27].

The HSR acquisitions were performed on the mosaic surface in two perpendicular directions with a step of 5 mm. The antenna was moved upon a transparent plexiglass sheet, in order to keep the manual acquisition as precise as possible and in order to avoid the direct physical contact to the surface. 

#### 2.2.2. Digital Holographic Speckle Pattern Interferometry (DHSPI) and Stimulated Infrared Thermography (SIRT) Workstation

The simultaneous application of digital holographic speckle pattern interferometry (DHSPI) and stimulated infrared thermography (SIRT) is already a recognized combined approach for subsurface structural diagnostics of a wide range of cultural heritage objects [31,32,33,34,35,36,37,38,39,40]. Both methods are non-invasive, full-field, remote control and non-contact techniques, aimed to evaluate the structural condition of the examined objects. The digital holographic speckle pattern interferometry (DHSPI) is based on the hybrid geometry combination of holographic interferometry (HI) and electronic speckle pattern interferometry (ESPI) [40,41]. Through a simultaneous DHSPI–SIRT acquisition, complementary information on the subsurface structure is obtained: DHSPI provides immediate visualization of the surface micro-displacement revealing the underlying cause that it is usually a subsurface hidden discontinuity, while SIRT allows immediate visualization of thermal energy diffusion on the surface of decorated surfaces providing complementary information on the homogeneity in materials composition and their integrity.

**Figure 4:**
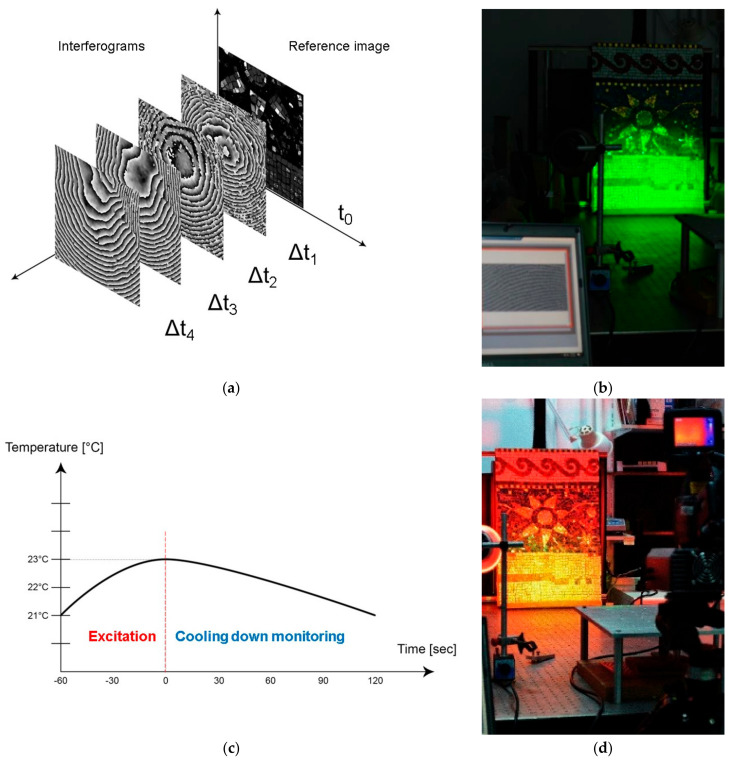
Scheme and photographic documentation of simultaneous acquisition procedure by digital holographic speckle pattern interferometry (**a,b**) and stimulated infrared thermography (**c,d**). Interferograms are acquired at regular intervals, which are set for the specific acquisition (each Δt indicates the time interval from the start of acquisition until the captured moment). Thermograms are acquired as a sequence of images at regular intervals set for the specific acquisition (1 s).

The holographic principle of DHSPI consists in recording phase variations of two mutually coherent laser beams, produced in the recording plane by a laser source of monochromatic light at the single wavelength of 532 nm: The object beam (called OB), reflected from the surface, interferes with the reference beam (called RB), which was not emitted onto the surface. The superposition of holographic wave fields gives rise to visible interferometric fringes, which indicate the out-of-plane displacement of the surface. Therefore, the data acquisition is based on an interferometric comparison of the two states of the surfaces under examination: The reference state and the excitation state. The white-light image is captured before the excitation and the subsequent acquisitions are performed with a defined regular interval after the excitation. The images are captured using a five-frame algorithm and each set of five images is compared to the first initial set, as shown in Figure 4a. The interferogram acquisition is coupled with the custom-built dedicated software allowing direct visualization of defect shape, size, and location through the identification of abnormal shape of fringe patterns [36]. The technique is directly quantitative through further processing with unwrapping algorithms. The magnitude of surface displacement, expressed in μm or fractions of micrometres, corresponds to the number of fringes multiplied by ½*λ* laser wavelength. Quantitatively, the displacement resolution (distance between two next pairs of interferometric fringes) is 266 nm (1/2 wavelength) [36]. The experimental set-up uses an Nd:YAG laser as a light source with the following characteristics: 250 mW at 532 nm, DPSS (diode pump solid state) of high spatial-temporal coherence with TEM:00 SLM (single longitudinal mode) and coherent length of 30 m. The high-resolution digital recording medium is a CCD detector with a pixel size of 4.4 μm. The detailed presentation of the system can be found in the relative literature references [31,32,33,34,35,36,37,38,39,40].

When DHSPI is coupled with SIRT, thermal excitation is used to reveal the hidden discontinuities by both techniques [40]. The SIRT measures and records variations in thermal radiation from the surface of an object in the infrared range, after induced thermal stress [42,43,44,45,46,47], as shown in Figure 4b. The resulting thermogram is a distribution map of the temperature on the surface of the object under investigation [47]. The photo-thermal signal depends on different parameters, including thermal conductivity, thermal emissivity, thermal diffusivity, temperature, surface colour and brightness, regularity and geometry of the surface. In addition, these parameters can be correlated to the characteristics that are of a strong interest in the wall decoration diagnostics: The presence of cracks, voids, the internal structure of the material, the progress of a physical and chemical transformation, etc. The detection system is constituted of an infrared camera working in a synchronous way with the excitation system (20° × 15°/0.3 m field of view, 1.1 mRad spatial resolution, 0.05 °C or 50 mK at 30 °C thermal sensitivity, 7.5 to 13 μm Spectral range). The measurement can be performed in the range of temperature from −40 °C to 120 °C.

**Figure 5:**
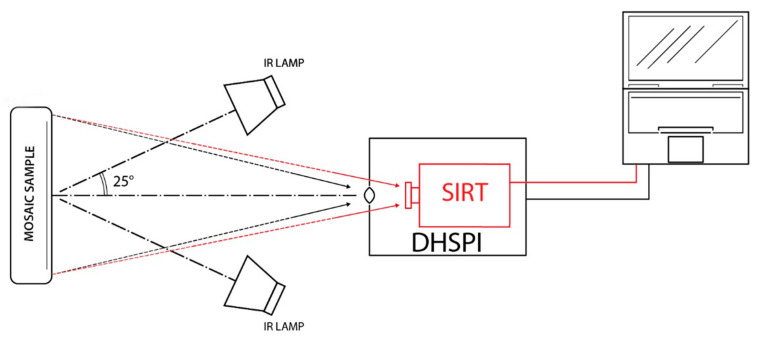
Simultaneous digital holographic speckle pattern interferometry (DHSPI) and stimulated infrared thermography (SIRT) laboratory acquisitions at the Holography Metrology Lab, IESL-FORTH. Scheme of the experimental workstation.

The DHSPI–SIRT workstation is illustrated in Figure 5 and comprises an excitation device (infrared lamps), a custom-built DHSPI set-up and an infrared camera, electronic and computing instrumentation for monitoring. Two infrared lamps of 175 W with 30° beam angle were placed symmetrically to the orthogonal axis of the object’s surface (from 20 to 45°), at the distance from 50 to 110 cm, in order to provide homogeneous thermal excitation onto the investigated surface. Several sensors connected to the same pc, in particular, a thermometer and a relative humidity meter, are recording the environmental RH/T values before the excitation and throughout the experiment.

The experimental procedure can be subdivided into three main steps: (1) Acquisition of reference values by DHSPI, thermal excitation for a specified duration, (2) simultaneous DHSPI and SIRT measurements in sequential recording during the cooling down process, (3) data post-processing, evaluation, and comparison. 

In this study, following preliminary experiments, six different thermal excitation values were selected: 20, 40, 60, 80, 120, 160 and 180 s. The interferograms and thermograms recording start always immediately after the end of the excitation loading. The reason is to capture the first and the highest surface reaction, which provides with the highest spatial frequency to upgrade the system ability to trace defects. The interval between captured interferograms by the DHSPI system was set to three seconds. and the SIRT sampling frequency was set to one second. The relaxation of a thermally excited object between the initial and the altered thermal values depends uniquely on the structural condition of the object. Preliminary tests provide the parameters of cooling down duration. In this study, the cooling down duration was in the range of 5–15 min. The sample was stored. and the tests were performed in the standard laboratory ambient conditions (temperature of 20–22 °C and relative humidity of 40–45%).

## 3. Results 

### 3.1. Results of HSR Acquisitions

**Figure 6:**
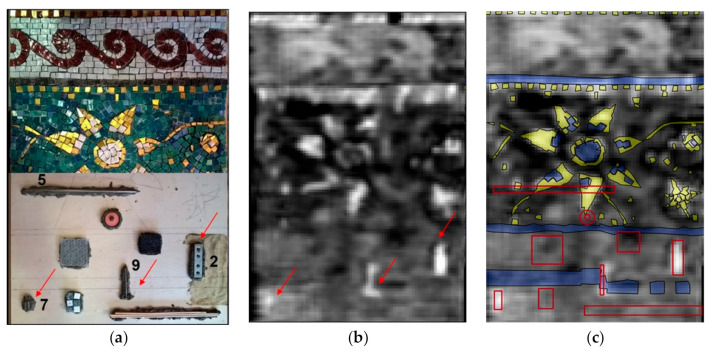
Buried defects and HSR result. (**a**) Defects in the subsurface structure and (**b**) HSR image at 6.7–6.8 GHz, where the red arrows indicate the location of the detected embedded metallic elements, (**c**) HSR image with the location of defects (contour in red), stone tesserae (blue) and golden leaf tesserae (yellow).

Since the surface height of the mosaic sample is variable (the difference between the lowest and the highest points is of 20 mm), the acquisitions were performed on six small areas (25 × 25 cm and 20 × 30 cm). The most appropriate depth value was selected for each acquisition area, in order to focus at the location of the known defects. Figure 6 shows an image of the six stitched small area acquisitions, acquired with the operating frequencies of 6.7 and 6.8 GHz, focused at 1.5–2.5 cm below the level of the decoration surface. 

The obtained HSR image reveals the known complex inhomogeneity of the custom-built sample. The stitches of mortar and the proportional differences in composition are not detectable by HSR technique since they do not provide sufficient contrast in dielectric permittivity. For cement-based mortars it depends on the initial water-to-cementitious ratio, fineness of the cement and other factors but usually, the dielectric constant is around ε_r_ = 2.2 [48]. The general inhomogeneity of the mosaic medium, detectable in the HSR image (Figure 6b,c) can be related to different factors (surface irregularity, mortar structure) on its surface or in the subsurface of the model. The reflections from the irregular surface dominate reflections from the subsurface buried targets. The strongest reflections in the HSR image correspond to the gold tesserae on the surface of the decoration layer and to the buried metallic elements (defects 2, 7 and 9) in the subsurface: A metallic steel plate, a steel nail, and a bent metallic wire. The reflections from the metallic objects are strong, due to the high contrast in dielectric permittivity (ε_r_ = 2.2 for the cement-based mortar and ε_r_ = infinite for the metal) [48]. The only buried metallic object (defect 5) is not detectable. Each grayscale holographic image shows a relative distribution of reflected signal intensities. Most probably, the defect 5 (metallic stick) is not detectable due to the dominance of reflections from the surface golden leaf tesserae. 

Comparison of the 3D virtual model of the mosaic (Figure 1c) to the HSR result (Figure 6b) allowed a better understanding of the influence of surface irregularity on the experimental results. It is, however, difficult or even impossible to discriminate between the other discontinuities detectable in the HSR image without the use of complementary techniques. 

### 3.2. Results of DHSPI Acquisitions 

**Figure 7:**
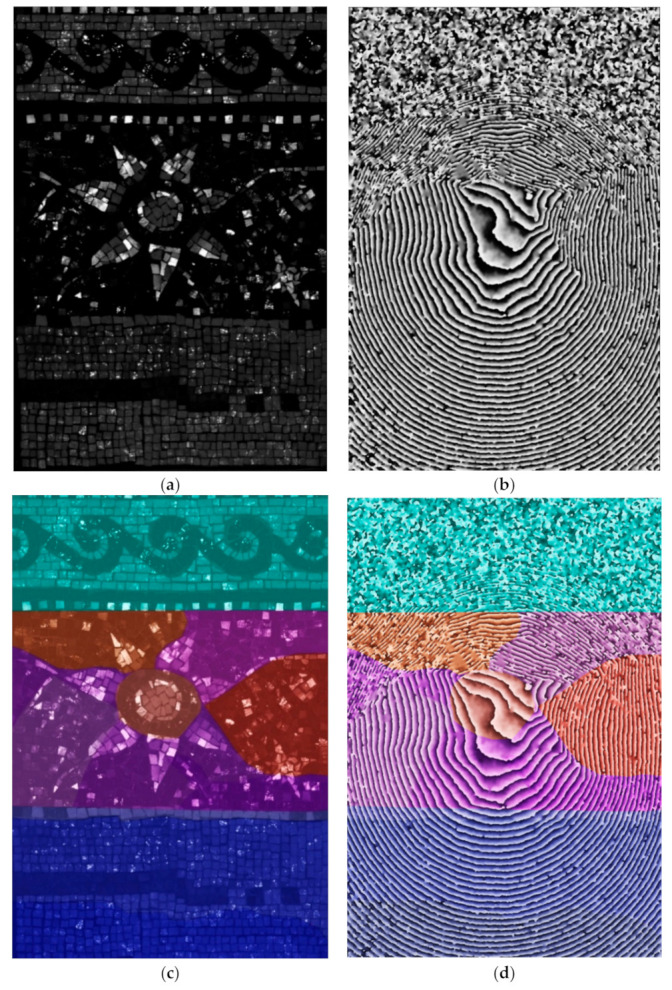
Full-field of total surface acquisition on the mosaic sample: (**a**) White light image, (**b**) interferogram at 49 s of cooling down monitoring with surface temperature Δt = 1.4 °C (80 s of excitation in a full-field acquisition), (**c**) overlay of the subsurface mortar structure scheme and the white light image, (**d**) overlay of the subsurface mortar structure and the interferogram.

Starting from Δt = 0.6 °C of thermal excitation for full-field acquisitions (lamps at the distance of 90 cm from the mosaic surface), the interferograms started to show clear differences in fringes pattern density and inclination on the mosaic surface Figure 7 shows the results at 49 s of cooling down after Δt = 1.4 °C surface temperature gradient. The examination of interferograms allowed immediate recognition of local areas with distinct but diverged morphology from the usual uniformity of fringes, as well as discontinuities formed by parallel dead-end fringes, shown in Figure 7b. Generally, the changes in fringes density are correlated to different properties in underlying materials (their composition and microstructure). The dead-end fringes may indicate the presence of cracks [32,33]. 

Figure 7d shows the documented scheme of mortar distribution overlaid on the interferogram captured at 49 s of cooling down monitoring at 1.4 °C surface temperature (80 s of excitation at a distance of 90 cm). The shown interferogram demonstrates changes, which predominantly match the documented stitches of the mortar portions.

**Figure 8:**
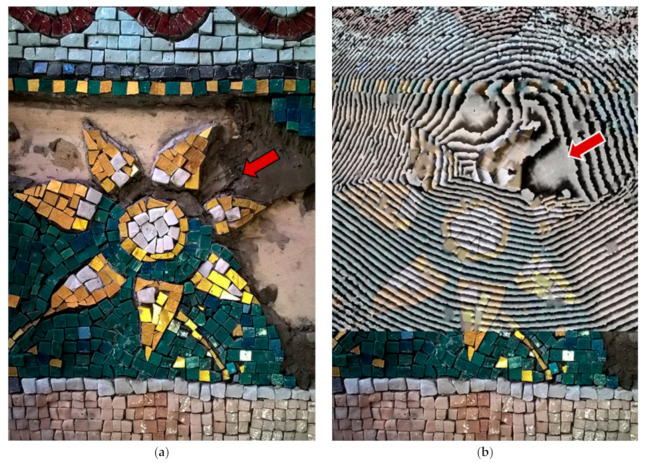
Recognition of the internal mortar structure in the mosaic sample: (**a**) Sample preparation in progress, detail of mortar application, (**b**) interferogram at 170 s after Δt = 3.1 °C thermal excitation (80 s of excitation in a detailed acquisition) on a completed mosaic model overlaid with the photographic documentation of the sample preparation.

In detailed acquisitions, the first differences in mortar structure were observed after 40 s of thermal excitation starting from the thermal gradient of 0.5 °C on the surface of the sample. Figure 8a shows the photographic documentation of the mosaic realization process (September 2016). Figure 8b shows an interferogram captured at 170 s after Δt = 3.1 °C (80 s of thermal excitation at a distance of 45 cm) on a completed mosaic model (March 2018).

**Figure 9:**
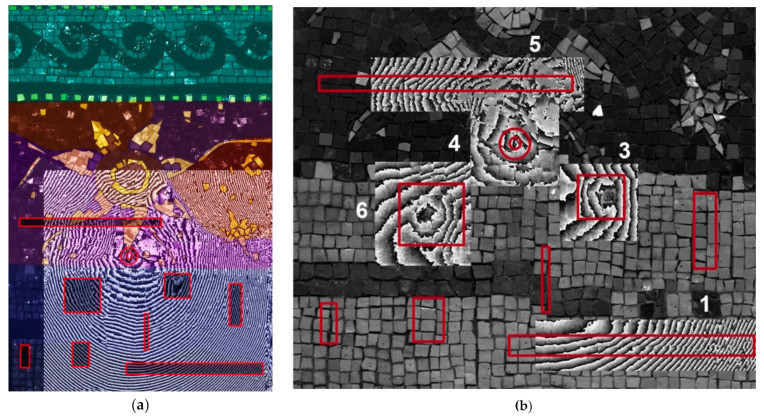
Results of the detailed DHSPI–SIRT acquisitions: (**a**) Overlay of complex sample inhomogeneity over the interferogram captured at 200 s after Δt = 2.1 °C thermal excitation, (**b**) summary of detected known defects in small area acquisitions. These defects include wooden sticks (defect 1) and synthetic sponge (defect 3) at 325 s after Δt = 3.8 °C, rubber ring (defect 4) at 207 s and steel stick (defect 5) at 345 s after Δt = 2.1°C thermal excitation, air bubble wrap (defect 6) at 944 s after Δt = 3.9° C thermal excitation.

A big detachment due to synthetic sponge (defect 3) was the first defect to be detected through open curves in the fringes pattern starting from approximately 120 s of cooling down monitoring after induced thermal impact Δt = 1.0 °C (60 s of IR excitation at a distance of 50 cm), as shown in Figure 9a,b. This pattern can be classified as corresponding to an internal crack or detachment [32,33]. The air bubble wrap (defect 6) induced an impact on the surface as open curves in the fringes pattern starting from 200 s after Δt = 1.4 °C thermal excitation (80 s of IR excitation at a distance of 50 cm). Other defects are also detectable, for example, the rubber ring (defect 4) as an irregular circular fringe within a complex morphology of fringes pattern and the steel stick (defect 5) as a change in the inclination of the curves (Figure 9a,b). The defects simulating detachments (i.e., synthetic sponge) appear on the interferograms as open curves after lower thermal excitation (starting from Δt = 1.4 °C) and as closed curved fringes after higher thermal excitation (at Δt = 2.1 °C and Δt = 3.9 °C). According to the fringes pattern classification [32,33], these known defects are recognized as an internal detachment or void. The presence of a flat steel inox plate (defect 2) was not detected by the DHSPI system by full-field of overall surface acquisition or even local detailed area acquisitions. The thermal excitation induced on the defect in the subsurface did not induce sufficient deformation in the direction of the optical axis of the system, in order to provide an impact on the mosaic surface. 

### 3.3. Results of SIRT Acquisitions

**Figure 10:**
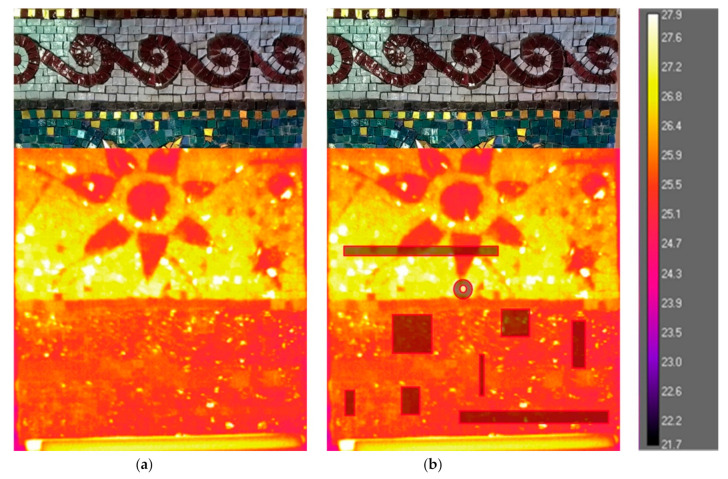
SIRT results: (**a**) Thermal image at 1st second after Δt = 1.2 °C (180 s of excitation with IR lamps at the distance of 110 cm), showing the position of the acquisition area with the regards to the whole surface area of the mosaic sample, (**b**) the same image showing the location of the embedded defects.

Differences in the surface temperature distribution, observed on the thermal images, can be related to both surface and subsurface characteristics of the mosaic [46,49,50]. As mentioned before, the decoration layer consists of marble tesserae (thermal conductivity k = 3.14 W/(mK) [46], glass paste tesserae (thermal conductivity is to be estimated due to variable contents of lead and other metal oxides) and metal leaf under a thin layer of glass (thermal conductivity is to be estimated). The tesserae are of different brightness, colour and are placed at different angles. Some of the tesserae act as a mirrored surface, creating a shimmering effect of reflections on the thermogram, as shown in Figure 10. The technique did not reveal the differences in mortar structure. The presence of buried objects is hardly detectable, e.g., detachment due to synthetic sponge (defect 3) and wooden sticks (defect 1) or not detectable, e.g., steel inox plate (defect 2) by visual observation of thermal images (Figure 10a,b). 

**Figure 11:**
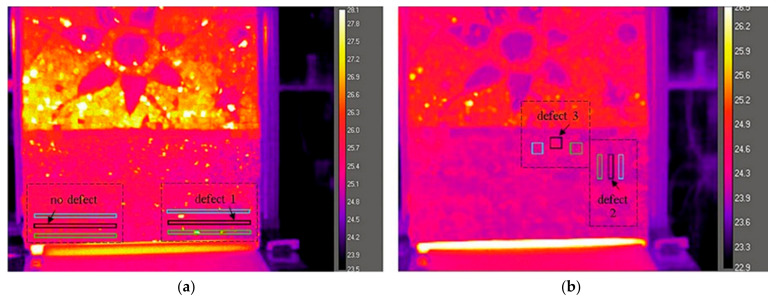
The position of the ROIs above the defect and in the surrounding areas: (**a**) Thermal distribution between the ROIs on the right side of the sample (defect 1) is compared to symmetrically placed ROIs on the left side of the sample (no defect), (**b**) thermal distribution between the ROIs for the defect 2 and the defect 3.

The analysis of IR thermography data was carried out on the results of the SIRT acquisition, presented in Figure 10. The tests were implemented with the help of dedicated software to evaluate the detectability of the wooden sticks (defect 1), metallic plate (defect 2) and synthetic sponge (defect 3). The thermal behaviour in the regions with or without defects underneath the surface is expected to be different. Therefore, several regions of interest (ROI) on the surface of the mosaic were selected and contoured on the thermal images. For each of the analysed defects, three ROIs of equal form and dimensions were selected: One covering the area with the defect underneath the mosaic surface and two covering the areas nearby without the defect underneath the mosaic surface. For defect 1, three equal rectangular ROIs are of 3 × 100 mm each: One covering the embedded wooden sticks and two the surrounding areas (higher and lower from the defect location). For defect 2, each ROI is of 10 × 50 mm: one covering the embedded steel plate and two equal ones covering the surrounding areas on the right and on the left side from the defect. For defect 3, each ROI is of 30 × 30 mm: One covering the embedded synthetic sponge and two equal surrounding areas on the right and on the left side from the defect. Additional three rectangular ROIs, equal in dimensions and form to the ROIs of the defect 1 on the right side of the mosaic surface, are positioned symmetrically on the left side of the mosaic surface. They do not cover embedded defects and are used as a reference for the study of defect detection. The position of the ROI for each of the analysed defects is shown in Figure 11.

The average temperature values per each analysed area (ROI), called mean temperature values, were calculated during the cooling down process after the acquisition with Δt = 1.2 °C on the surface temperature (180 s of excitation) At the 1st second of excitation, each of the three defected areas (1, 2 and 3) shows higher mean temperature values than their surrounding areas. The differences are detectable for defect 1 and 3 (wooden sticks and synthetic sponge) and not detectable for defect 2 (steel plate) by visual observation of thermal images. The temperature values of each defected area and the thermal differences with the surrounding areas at the start and at the end of cooling down monitoring after Δt = 1.2 °C thermal excitation are reported in Table 2, Table 3 and Table 4. 

Figure 11a shows the position of the analyzed ROIs for the comparison of mean temperature values over the areas inside and outside the defect 1. The analysis was also performed on three equal ROIs, placed symmetrically on the left side of the sample, as shown in Figure 11a. These ROIs cover partially mosaic tiles and of a metallic clip underneath the decoration layer, not detectable through the surface temperature differences. 

**Figure 12:**
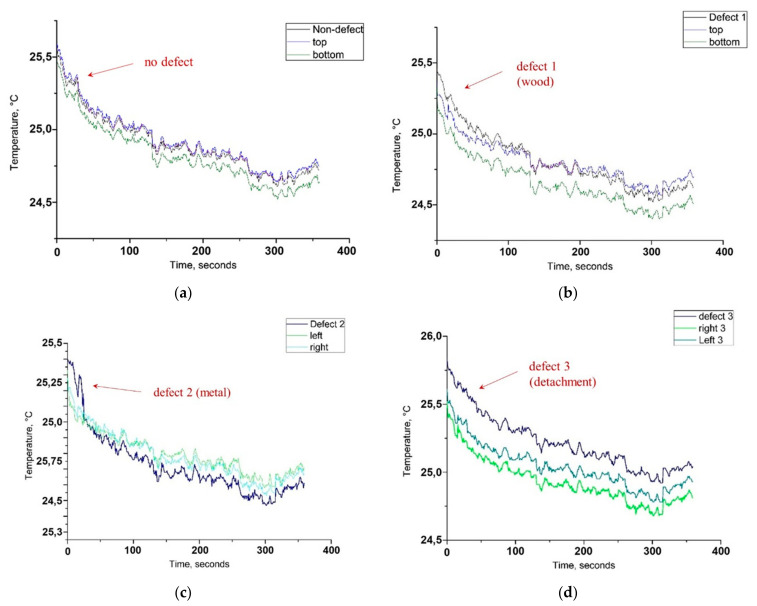
Defect detection by the means of SIRT data analysis. Comparison of mean temperature values per area (ROI) located over the defect, embedded in the subsurface, and two equal areas located on the surrounding areas: (**a**) Three ROIs equal in form and dimension to the defect 1, which cover non-defected areas, used as a reference of expected thermal distribution, (**b**) three ROIs equal in form and dimension to the defect 1, which cover the area over the defect, and two surrounding areas, (**c**) three ROIs equal in form and dimension to the defect 2, which cover the area over the defect and two surrounding areas, (**d**) three ROIs equal in form and dimension to the defect 3, which cover the area over the defect and two surrounding areas.

On the left side of the mosaic model, without buried wooden sticks, the temporal temperature plots show a distribution of values, expected for a non-defected area of the mosaic (Figure 12a). The ROIs correspond mostly to the non-defected area, covering only partially the buried mosaic tiles and a metallic clip, not detectable by visual observation of thermal images. On the right side of the mosaic, with the buried wooden sticks hidden in the subsurface, the higher mean temperature at 1st second after excitation is detected in the defected area (25.47 °C), compared to the surrounding areas (25.37 and 25.38 °C respectively), as shown by the graph in Figure 12b. The temperature inside the ROI covering the wooden sticks equalises with the values of the surrounding areas at 110 s after excitation. At the end of the cooling down monitoring the distribution of values reaches the values, similar to the left side of the mosaic, as expected for a non-defected area. The defect is detected through higher surface temperature above the embedded wooden sticks, which can be explained by the lower thermal conductivity of the wood, compared to the thermal conductivity of the cement mortar binding medium (0.05–0.14 W/(mK) for different types of wood and 1.4–1.75 W/(mK) for the cement mortar) [46,50]. 

As shown by the temporal plot (Figure 12c), at the 1st second after thermal excitation, the area upon the defect 2 (a stainless steel plate) shows slightly higher mean temperature values than its surrounding areas (difference of 0.2 °C), which is not detectable by visual observation of thermal images. This difference is maintained during the first 30 s of cooling down monitoring, afterwards, a temperature equalisation of the surface occurs and, subsequently, starting from 92 s, the temperature values become inverted, with a very low difference (0.1 °C after 300 s cooling down monitoring). 

**Figure 13:**
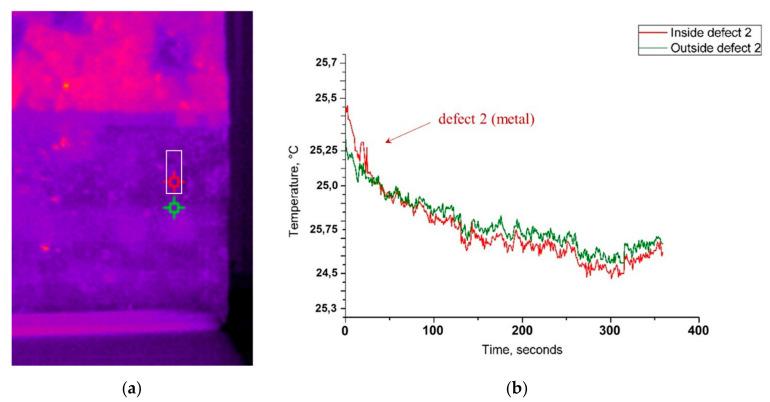
Temporal plots for thermal point analysis: (**a**) Position of the analysed points 3 · 3 pixel each (red point over the buried defect 2 and green point without any buried defect underneath, the white rectangle shows the location of the metallic plate), and (**b**) graph showing point temperature measurements over time.

In order to furtherly analyse the thermal behaviour of the buried metallic object (defect 2), additional thermal plots for two points on the surface of the mosaic were calculated and plotted (each point equals 3 × 3 pixel), as shown in Figure 13a. One point is located on the mosaic surface covering the embedded metallic object underneath (inside defect 2) and the other point is located in its proximity without the embedded metallic object underneath (outside defect 2). Both points are located on the homogeneous surface of the glass paste tesserae. As shown in Figure 13b, at 1st second after thermal excitation, the point temperature within the defected area 2 (steel plate) is 0.17 °C higher than of the point outside it (25.45 and 25.27 °C respectively). The temperature inversion occurs at 31 s after excitation and the low difference remains persistent until the end of cooling down monitoring, with the maximum Δt = 0.12 °C. It can be explained by the fact that the metal due to its higher thermal conductivity (k = 16 W/(mK)) is a good thermal conductor, it heats faster and cools down faster than the surrounding medium, characterized by lower thermal conductivity (k = 1.75 W/(mK) for the cement-based mortar [46,50]. 

The defect 3 (synthetic sponge simulating a thick detachment) is detectable by simple visual observation of thermal images, due to the higher temperature value, common for voids or detachments. However, in the temporal graphs (Figure 12d), unlike the wooden sticks or steel plate, the thermal difference on the surface remains persistent. An exchange in temperature values between the defected and non-defected areas is yet not observed until the end of cooling down monitoring at 360 s. It can be explained by the fact that the thermal conductivity values for the synthetic sponge, which contains predominantly air and plastic material are low (k = 0.026 W/(mK) and k = 0.2–0.3 W/(mK), respectively) [46,50]. This object acts as a thermal insulator. The thermal energy remains blocked inside the sponge and longer monitoring time may be needed for the temperature between the defect and its surroundings to be equalized, as shown by the temporal plot in Figure 12d.

## 4. Discussion 

The three techniques, operating with different principles, provided complementary information on the embedded defects and subsurface homogeneity of the custom-built mosaic model. The tests were performed as non-contact full-field acquisitions by DHSPI and SIRT and as detailed contact acquisitions by HSR. 

The DHSPI system showed its high potentiality for the study of subsurface medium homogeneity. By observation of the interferograms over time, it was possible to reconstruct the known internal structure of mortar and differentiate between distinct inlays, applied one after another. The system was capable to reveal the differences in the proportions of mortar’s composition and in the way it had been applied (flattened, regular vs. rapid, and irregular), as well as the stitches between different parts. Observation of fringes morphology over time in DHSPI results allowed localizing the defects that simulate the presence of adhesion loss, detachments, and voids. The thick detachment was also detectable by simple observation of SIRT images. Regarding the hidden metallic objects, the steel plate was not detected: Neither by the examination of interferograms pattern nor by visual observation of thermal images. Thermal excitation did not provoke vertical displacement in the metal sample, therefore, no impact on the surface could be detected by the DHSPI. The metal plate could be revealed subsequently by post-processing of thermal data by the means of dedicated software. 

The steel plate and the steel nail were, however, immediately detectable by the third complementary technique–HSR. This method showed high potentiality in the detection of high contrasts in dielectric permittivity when the object width in a planar surface is bigger than the acquisition step of the technique. The signal is reflected from a metal interface without any further penetration inside the medium, which represent, from one side, an advantage in the detection of hidden metal elements and a disadvantage, from the other side, during acquisitions upon signal reflective surfaces. This signal does not penetrate underneath the golden tesserae layer, which is a strong limitation for the field of Byzantine wall mosaics diagnostics. The signal is strongly attenuated through superficial reflections from the irregular mosaic tesserae surface with high metal oxides content (up to 20%). The detectability of known embedded defects and unintentionally created defects (cracks, damages, etc.) in the given laboratory experimental study is summarized in Table 5.

## 5. Conclusions

As shown by the first laboratory experimental results, the three techniques, operating with different principles, provided complementary information on the subsurface homogeneity and presence of known anomalies within the custom-built mosaic model. Each of the tested methods demonstrated certain potentialities and limitations when applied to the decorated surface of a mosaic model in this experimental study. 

The holographic subsurface radar (HSR) showed the following limitations: Need of physical contact to the surface, results are highly influenced by surface geometry and material transparency on the microwave signal, absence of penetration under the golden leaf tesserae, possible errors during manual acquisition, discrimination between the reflections from the irregular surface and the reflections from the subsurface target is difficult. The proved key advantage of this technique lies, however, in the detection of metal elements larger than the resolution step of the used instrument (0.5 cm) even under irregular glass paste or stone mosaic tesserae surface. 

The digital holographic speckle pattern interferometry (DHSPI) showed limitation in the detection of micro-displacement other than the z sensitivity direction of the optical axis of the system, the elements without much impact on the examined surface were not detected (e.g., the thin metal plate in the specific laboratory case study producing deformation only to x, y direction). The main advantages include its full-field, remote and non-contact method of operation, applicability to irregular and non-planar surfaces and good penetration underneath the metal leaf and other types of tesserae, immediate detailed information about detachments, cracks, and differences in the medium structure (mortar). The main disadvantage is the vibration sensitivity, which demands steady optical table and to avoid noises during data acquisition.

The stimulated infrared thermography (SIRT) showed the following limitations: Non-visibility through the glass, difficulties to distinguish between different objects with similar temperature, results are influenced by surface reflectivity and colour. The advantages of this technique include immediate non-contact detection of contrasts in thermal conductivity, the good potentiality for the study of shallow depth detachments and voids. Further SIRT tests, using longer thermal excitation times, should be implemented, enabling in such a manner the diffusion of a greater amount of energy into the interior of the tested mosaic model. In this regard, the use of most advanced procedures of active thermography [51,52] could possibly overcome the detectability limitations observed in this study, which is mainly attributed to the limited heat excitation energy induced into the mosaic model. 

**Figure 14:**
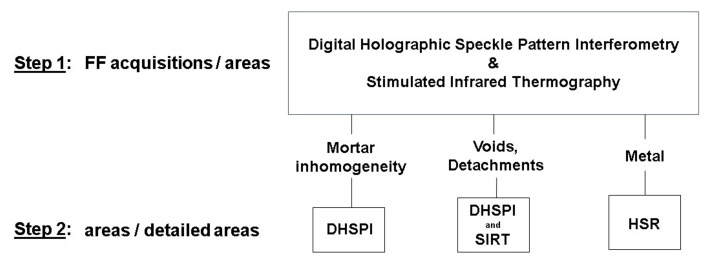
The recommended workflow of a combined non-invasive approach to the mosaic diagnostic study, based on the first laboratory experimental results. FF–full field, DHSPI–digital holographic speckle pattern interferometry, SIRT–stimulated infrared thermography, HSR–holographic subsurface radar.

On the basis of the results obtained in this experimental study, we propose a combined non-invasive approach to the mosaic diagnostic study following a procedure illustrated in Figure 14. 

The results of the first laboratory experimental results show that non-contact full-field techniques DHSPI and SIRT can be applied in combination as first pre-screening tools in full field (FF) or larger areas acquisitions for mapping the areas of interest in the subsurface of mosaics for further detailed acquisitions. These techniques can be applied in-situ on real mosaic case studies and, subsequently, depending on the available documentation, conservation issues and the first full-field results, the selection of methods for further detailed area acquisitions may be different. If contact to the mosaic surface is allowed, and if the area of interest lies underneath non-metallic tesserae, we recommend testing HSR technique for the detection of metallic elements in the mosaic subsurface (nails, anchors and other fixation elements). When contact to the surface is not allowed, but a minimal thermal excitation is acceptable, SIRT can be tested for the detection of metallic elements. When the presence of detachments or cracks is suspected, further full-field monitoring and detailed acquisitions by DHSPI–SIRT are highly recommended. Furthermore, the DHSPI results on a mosaic model with known mortar structure showed the high potentiality of the DHSPI technique in the detection of mortar inhomogeneities underneath the mosaic decoration layer. It should be further tested in-situ as a non-invasive method for differentiation between original and reshuffled areas in historical mosaics. The complementarity of data, obtained through the combination of evaluated techniques on the known anomalies in a mosaic model, indicates strong potentiality of further development of the proposed non-invasive approach to the in-situ subsurface analysis of ancient wall mosaics.

## Figures and Tables

**Table 1:** Location depth of the embedded defects.

Defect	Location Depth (mm)
wooden sticks (defect 1), steel plate (defect 2), air bubble wrap (defect 6) and metallic clip (defect 7)	20
synthetic sponge (defect 3), rubber ring (defect 4), metallic stick (defect 5), marble and glass tiles (defect 8) and steel nail (defect 9)	15

**Table 2:** Mean temperature per equal area (ROI) inside and outside the defect 1.

Defect 1 (Wood)	Max. T1 (°C) During the Cooling Down Monitoring	Min. T2 (°C) During the Cooling Down Monitoring	ΔTmax (T1–T2)
Defected area	25.47	24.56	0.91
Non-defected top	25.38	24.58	0.80
Non-defected bottom	25.37	24.41	0.96

**Table 3:** Mean temperature values per equal area (ROI) inside and outside the defect 2.

Defect 2 (Steel)	Max. T1 (°C) During the Cooling Down Monitoring	Min. T2 (°C) During the Cooling Down Monitoring	ΔTmax (T1–T2)
Defected area	25.40	24.48	0.92
Non-defected left	25.33	24.58	0.75
Non-defected right	25.28	24.56	0.72

**Table 4:** Mean temperature values per equal area (ROI) inside and outside the defect 3.

Defect 3 (Plastic and Air)	Max. T1 (°C) During the Cooling Down Monitoring	Min. T2 (°C) During the Cooling Down Monitoring	ΔTmax (T1–T2)
Defected area	25.83	24.98	0.85
Non-defected left	25.61	24.79	0.82
Non-defected right	25.52	24.69	0.83

**Table 5:** Detectability of the defects by the experimental laboratory results.

Known Defects and Discontinuities	DHSPI	SIRT	HSR
Differences in composition and stitches of mortar	**detectable**	no detectable	only general inhomogeneity
(1) wooden sticks	**detectable**	**detectable**	not detectable
(2) steel plate	not detectable	**detectable through data processing**	**detectable**
(3) semi-rigid synthetic sponge	**detectable**	**detectable**	not detectable
(4) rubber ring	**detectable**	**detectable**	not detectable
(5) steel stick	detectable in detailed acquisitions	not detectable	not detectable
(6) air bubble wrap (shallow detachment)	**detectable**	not detectable	not detectable
(7) metal clip	not detectable	not detectable	**detectable**
(8) steel nail	not detectable	not detectable	**detectable**
(9) glass and marble tiles	not detectable	not detectable	not detectable

## References

[1] Farneti M. (1993). Glossario Tecnico-Storico del Mosaico: Con Una Breve Storia del Mosaico.

[2] Fiorentini Roncuzzi I., Fiorentini E. (2002). Mosaic: Materials, Techniques and History.

[3] Henig M., Ithaca N.Y. (1983). A Handbook of Roman Art: A Comprehensive Survey of All the Arts of the Roman World.

[4] Hutter I. (1988). Early Christian and Byzantine Art (History of Art and Architecture).

[5] Demus O. (1948). Byzantine Mosaic Decoration: Aspects of Monumental Art in Byzantium.

[6] Cléveno D., Degeorge G. (2000). Ornament and Decoration in Islamic Architecture.

[7] Fiorentini Roncuzzi I. (1971). Arte e Tecnologia nel Mosaico.

[8] Mellentin Haswell J. (1973). Manual of Mosaic.

[9] Ben Abed A., Demas M., Roby T. (2008). Lessons Learned: Reflecting on the Theory and Practice of Mosaic Conservation.

[10] Vandini M., Fiore C. (2002). Teoria e Pratica per la Conservazione del Mosaico.

[11] Μουρικη Ντ (1985). Τα ψηφιδωτα της νεας Μονης Χιου (Διτομο).

[12] Von Landsberg D. (1992). The history of lime production and use from early times to the industrial revolution. Zement-Kalk-Gips.

[13] Schirripa S.G., Ambrosini D., Paoletti D. (1998). Optical methods for mosaic diagnostics. J. Opt..

[14] Moropoulou A., Avdelidis N.P., Delegou E.T., Aggelakopoulou E., Karoglou M., Haralampopoulos G., Griniezakis S., Koui M., Karmis P., Aggelopoulos A. (2000). Investigation for the compatibility of conservation interventions on Hagia Sophia mosaics using NDT techniques. J. Eur. Study Group Phys. Chem. Biol. Math. Tech. Appli. Archaeol..

[15] Martinho E., Dionísio A. (2014). Main geophysical techniques used for non-destructive evaluation in cultutal built heritage: A review. J. Geophys. Eng..

[16] Marcaida I., Maguregui M., Morillas H., Prieto-Taboada N., Veneranda M., Fdez-Ortiz de Vallejuelo S., Martellone A., De Nigris B., Osanna M., Madariaga J. (2019). In situ non-invasive multianalytical methodology to characterize mosaic tesserae from the House of Gilded Cupids, Pompeii. Herit. Sci..

[17] Artioli G. (2010). Scientific Methods and Cultural Heritage: An. Introduction to the Application of Materials Science to Archaeometry and Conservation Science.

[18] Angelo Orsoni Furnace Webite. https://www.orsoni.com.

[19] Verità M., Moldi C. (1996). Mosaico vitreo e smalti: La tecnica, i Materiali, il degrado, la conservazione. I Colori Della Luce: Angelo Orsoni e l’arte del Mosaico.

[20] Doremus R.H. (1994). Glass Science.

[21] Razevig V.V., Ivashov S.I., Vasiliev I.A., Zhuravlev A.V., Bechtel T., Capineri L., Falorni P. RASCAN Holographic Radars as Means for Non-Destructive Testing of Buildings and Edificial Structures. Proceedings of the Structural Faults and Repair-2010.

[22] Ivashov S.I., Makarenkov V.I., Masterkov A.V., Razevig V.V., Sablin V.N., Sheyko A.P., Tchapourski V.V., Vasiliev I.A. Concrete Floor Inspection with Help of Subsurface Radar. Proceedings of 6th Meeting Environmental and Engineering Geophysics.

[23] Ivashov S., Razevig V., Sheyko A., Vasilyev I., Zhuravlev A., Bechtel T. Holographic Subsurface Radar Technique and its Applications. Proceedings of the 12th International Conference on Ground-Penetrating Radar, GPR 2008.

[24] Capineri L., Falorni P., Borgioli G., Bulletti A., Valentini S., Ivashov S., Zhuravlev A., Razevig V., Vasiliev I., Paradiso M. (2009). Application of the RASCAN Holographic Radar to Cultural Heritage Inspections. Archaeol. Prospect..

[25] Capineri L., Falorni P., Ivashov S., Zhuravlev A., Vasiliev I., Razevig V., Bechtel T., Stankiewicz G. (2009). Combined Holographic Subsurface Radar and Infrared Thermography for Diagnosis of the Conditions of Historical Structures and Artworks. Eur. Geosci. Union Gen. Assem..

[26] Vasiliev I.A., Ivashov S.I., Makarenkov V.I., Sablin V.N., Sheyko A.P. (1999). RF band high resolution sounding of building structures and works. IEEE Aerosp. Electron. Syst. Mag..

[27] Ivashov S.I., Capineri L., Bechtel T., Taylor J.D. (2012). Holographic Subsurface Radar Technology and Applications. UWB Radar Applications and Design.

[28] Daniels D.J. (2004). Ground Penetrating Radar.

[29] Gabor D. (1948). A new microscopic principle. Nature.

[30] Razevig V.V., Zhuravlev A.V., Bugaev A.S., Chizh M.A., Ivashov S.I. Imaging Under Irregular Surface Using Microwave Holography. Progress in Electromagnetics Research Symposium—Fall (PIERS-FALL).

[31] Tornari V. (2018). On development of portable Digital Holographic Speckle Pattern Interferometry system for remote-access monitoring and documentation in art conservation. Strain 201.

[32] Tornari V., Tsigarida A., Ziampaka V., Kousiaki F., Kouloumpi E. (2017). Interference Fringe Patterns in Documentation on Works of Art: Application on Structural Diagnosis of a Fresco Painting. Am. J. Arts Des..

[33] Tornari V., Tsiranidou E., Bernikola E. (2012). Interference fringe-patterns association to defect-types in artwork conservation: An experiment and research validation review. Appl. Phys. A.

[34] Kosma K., Andrianakis M., Hatzigiannakis K., Tornari V. (2018). Digital holographic interferometry for cultural heritage structural diagnostics: A coherent and a low-coherence optical set-up for the study of a marquetry sample. Strain.

[35] Tornari V., Bernikola E., Nevin A., Kouloumpi E., Doulgeridis M., Fotakis C. (2008). Fully non-contact holography-based inspection on dimensionally responsive artwork materials. Sensors.

[36] Tornari V. (2007). Laser Interference-Based Techniques and Applications in Structural Inspection of Works of Art. Anal. Bioanal. Chem..

[37] Schirripa Spagnolo G., Guattari G., Grinzato E. Frescoes Diagnostics by electro-optic holography and infrared thermography. Proceedings of the 6th World Conference on NDT and Microanalysis in Diagnostics and Conservation of Cultural and Environmental Heritage.

[38] Tornari V., Bernikola E., Tsigarida N., Andrianakis M., Hatzigiannakis K., Leissner J. (2015). Preventive deformation measurements on cultural heritage materials based on non-contact surface response of model samples. Stud. Conserv..

[39] Bernikola E., Nevin A., Tornari V. (2009). Rapid initial dimensional changes in wooden panel paintings due to simulated climate-induced alterations monitored by digital coherent out-of-plane interferometry. Appl. Phys. A.

[40] Tornari V., Andrianakis M., Hatzigiannakis K., Kosma K., Detalle V., Bourguignon E., Giovannacci D., Brissaud D. (2016). Complimentarity of digital holographic speckle pattern interferometry and simulated infrared thermography for Cultural Heritage structural diagnostic research. Int. J. Eng. Res. Sci (IJOER).

[41] Vest C.M. (1979). Holographic Interferometry.

[42] Maldague X.P. (2001). Theory and Practice of Infrared Technology for Nondestructive Testing.

[43] Theodorakeas P., Cheilakou E., Ftikou E., Koui M. Passive and active infrared thermography: An overview of applications for the inspection of mosaic structures. Proceedings of the 33rd UIT, Italian Union Thermo-Fluid Dynamics Heat Transfer Conference.

[44] Jo Y.H. (2013). Study on Applicability of Passive Infrared Thermography Analysis for Blistering Detection of Stone Cultural Heritage. J. Conserv. Sci..

[45] Avdelidis N.P., Koui M., Ibarra-Castanedo C., Maldague X. (2007). Thermographic studies of plastered mosaics. J. Infrared Phys. Tech..

[46] Carlomagno G.M., Cardone G. (2010). Infrared Thermography for convective heat transfer measurements. J. Exp. Fluids.

[47] Ibarra-Castanedo C., Gonzalez D., Klein M., Pilla M., Vallerand S. (2004). Infrared image processing and data analysis. Infrared Phys. Tech..

[48] Von Hippel A.R. (1954). Dielectric Materials and Applications. The International Critical Tables.

[49] Incropera F., De Witt D. (1990). Introduction to Heat Transfer.

[50] Young H.D. (1992). University Physics.

[51] Laureti S., Silipigni G., Senni L., Tomasello R., Burrascano P., Ricci M. (2018). Comparative study between linear and non-linear frequency-modulated pulse-compression thermography. Appl. Opt..

[52] Laureti S., Mlekmohammadi H., Burrascano P., Hutchins A.D., Senni L., Silipigni G., Maldague X.P.V., Ricci M. (2018). The use of pulse-compression thermography for detecting defects in paintings. NDT E Int..

